# Visceral, Neural, and Immunotoxicity of Per- and Polyfluoroalkyl Substances: A Mini Review

**DOI:** 10.3390/toxics13080658

**Published:** 2025-07-31

**Authors:** Pietro Martano, Samira Mahdi, Tong Zhou, Yasmin Barazandegan, Rebecca Iha, Hannah Do, Joel Burken, Paul Nam, Qingbo Yang, Ruipu Mu

**Affiliations:** 1 The Basic Sciences Department, University of Health Sciences and Pharmacy in St. Louis, St. Louis, MO 63110, USA; 2 Cooperative Research, College of Agriculture, Environmental and Human Sciences, Lincoln University of Missouri, Jefferson City, MO 65101, USA; samira.mahdi183@my.lincolnu.edu (S.M.); barazandegany@lincolnu.edu (Y.B.); 3 Department of Civil Engineering, Missouri University of Science and Technology, Rolla, MO 65409, USA; 4 Department of Chemistry, Missouri University of Science and Technology, Rolla, MO 65409, USA

**Keywords:** PFAS exposure, public health, organ toxication, immune dysfunction, carcinogenic risks

## Abstract

Per- and polyfluoroalkyl substances (PFASs) have gained significant attention due to their widespread distribution in the environment and potential adverse health effects. While ingestion, especially through contaminated drinking water, is considered the primary route of human exposure, recent research suggests that other pathways, such as inhalation and dermal absorption, also play a significant role. This review provides a concise overview of the toxicological impacts of both legacy and emerging PFASs, such as GenX and perfluorobutane sulfonic acid (PFBS), with a particular focus on their effects on the liver, kidneys, and immune and nervous systems, based on findings from recent in vivo, in vitro, and epidemiological studies. Despite the transition to PFAS alternatives, much of the existing toxicity data focus on a few legacy compounds, such as perfluorooctanoic acid (PFOA) and perfluorooctane sulfonate (PFOS), which have been linked to adverse immune outcomes, particularly in children. However, evidence for carcinogenic risk remains limited to populations with extremely high exposure levels, and data on neurodevelopmental effects remain underexplored. While epidemiological and experimental animal studies supported these findings, significant knowledge gaps persist, especially regarding emerging PFASs. Therefore, this review examines the visceral, neural, and immunotoxicity data for emerging PFASs and mixtures from recent studies. Given the known risks from well-studied PFASs, a precautionary principle should be adopted to mitigate human health risks posed by this large and diverse group of chemicals.

## 1. Introduction

Per- and poly-fluoroalkyl substances (PFASs) were discovered unintentionally and quickly found broad applications due to their unique ability to repel water and oil and resist heat [1]. However, despite their technological benefits, scientific evidence has raised significant concerns about their environmental and health impacts.

PFASs were originally developed in 1938 by scientists in a lab, and the two most recognized PFASs in industry were introduced in the 1940s: DuPont’s perfluorooctanoic acid (PFOA), used to create Teflon and as a byproduct of many other processes, and 3M’s perfluorooctane sulfonate (PFOS), used in water repellent, firefighting foam, and semiconductor devices [2]. Since then, PFASs have been used extensively, allowing scientists to study their associated health impacts. More than 1000 studies, including some commissioned by the military, have been conducted [3]. Even manufacturers like DuPont and 3M carried out studies revealing the potential adverse health effects of PFASs in both animals and humans [4,5].

PFASs are a diverse group of thousands of manmade chemicals, characterized by their strong carbon–fluorine bonds, one of the strongest in nature [1]. These bonds give PFASs unique properties including oil and water resistance, durability, and the ability to withstand degradation [6]. Consequently, PAFSs can be seen in a variety of consumer products such as nonstick cookware, stain-resistant and weatherproof fabrics, and food packaging due to its oil- and water-resistant properties [7]. Other than their application in the food and agriculture sectors, they are used as surfactants in aerospace, construction, electronics, and firefighting foams [8].

In 2021, the Organization for Economic Co-operation and Development (OECD) specified in its revised definition that PFASs are fluorinated substances containing at least one fully fluorinated methyl or methylene carbon atom without any H/Cl/Br/I atom attached [9]. Due to their extraordinary chemical stability, once exposed to the human body, water, or the environment, they will persist indefinitely.

The prevalence of PFASs indicates that many people have been exposed to these chemicals. Studies confirmed by the Centers for Disease Control and Prevention (CDC) have found some form of PFASs in the blood of 95% of Americans [10]. These substances are not readily removed from the blood and have the potential to accumulate in visceral organs, where they may interfere with cellular function, potentially in ways that could lead to serious health deterioration for humans. Research has continuously focused on understanding the mechanisms through which PFAS exposure can affect different systems in human body [11,12].

**Figure 1:**
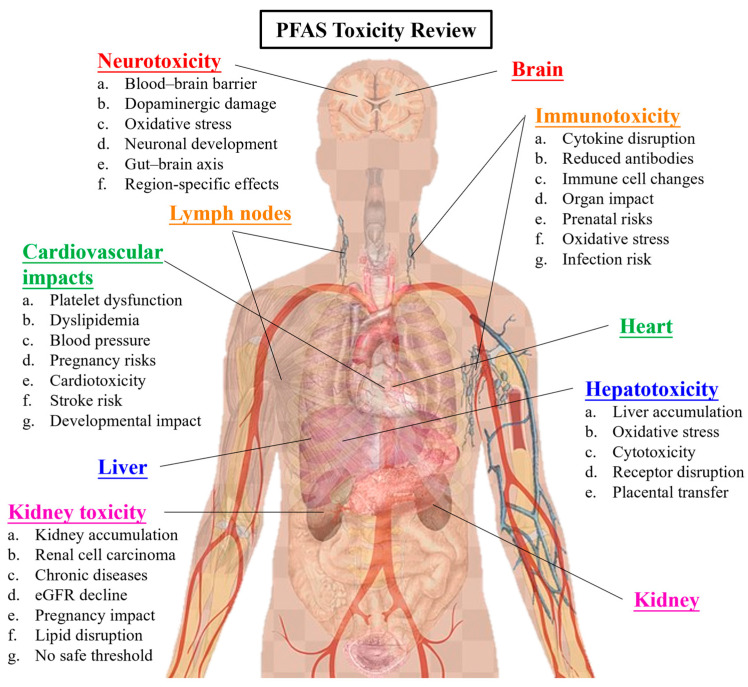
A schematic diagram of the covered PFAS toxicities in this mini review. Base elements adapted from Freepik (https://www.freepik.com (accessed on 30 December 2024)), used under the Freepik Free License with attribution. Final illustration created and modified by the author.

**Figure 2:**
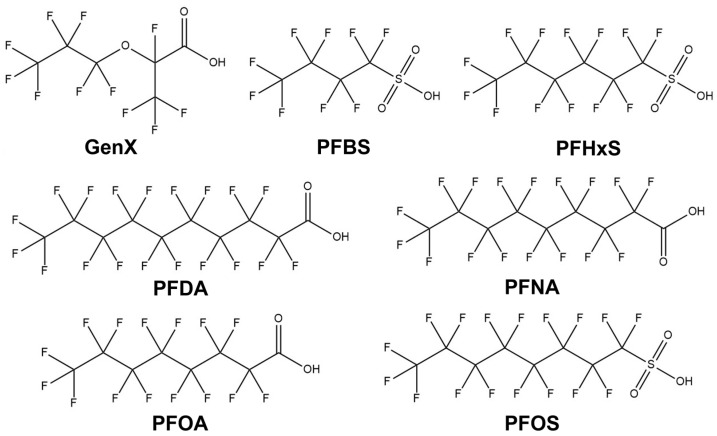
Chemical structures of selected per- and polyfluoroalkyl substances (PFASs). These compounds differ in chain length and functional groups, which influence their environmental persistence and toxicity profiles.

PFASs have become ubiquitous in the environment [13], and their presence has been documented in water [14,15], soil [16,17], and the atmosphere [18,19,20], as well as in various organisms, including humans [21,22], marine animals [23,24], birds [25,26], and plants [27,28]. Despite comprising a group of over 6000 chemicals [29] with diverse applications over the past seven decades [30], assessing the individual and synergistic impacts of PFASs on human health remains challenging. Furthermore, most PFASs exhibit low elimination rates and long half-lives in the human body [31,32]. PFAS half-lives vary widely depending on the chemical structure and biological matrix, ranging from several days (e.g., PFBS) to over 5 years (e.g., PFOS and PFOA) in human serum [33,34]. Epidemiological studies have demonstrated that exposure to PFASs is associated with various health risks [35], including hypercholesterolemia [36], neurotoxicity [37], nephrotoxicity [38], hepatotoxicity [39], immunotoxicity [40], cardiovascular disease [41], carcinogenesis [42], reproductive toxicity [43], etc. Although substantial progress has been made in understanding the effects of PFASs on animal and human health, significant knowledge gaps still exist regarding the mechanisms of PFAS toxicity and their broader implications. Therefore, this review aims to consolidate the most recent scientific insights into PFAS toxicological impacts on human health (as summarized in Figure 1) and explore strategies for advancing knowledge on PFAS toxicity. Figure 2 illustrates the chemical structures of selected PFAS discussed in this mini review.

## 2. Visceral Toxicity

The toxic effects of PFASs on the visceral system have been extensively studied, where prolonged exposure to specific PFAS compounds has been linked to hepatotoxicity, nephrotoxicity, and reproductive toxicity in both animal models and human studies [44,45,46,47,48,49]. These substances tend to accumulate in visceral organs, interfering with cellular processes and potentially resulting in severe health outcomes [12,50].

### 2.1. Hepatotoxicity

The liver is widely recognized as a primary organ for PFAS accumulation, largely due to its role in synthesis of human serum albumin (HSA), to which PFASs readily bind in plasma [51,52,53]. Consequently, the impact of PFAS exposure on hepatic function has become a central focus in toxicological research. Studies in this field predominantly involve analyzing human serum samples from contaminated regions, conducting in vitro experiments using hepatocyte cell lines, and utilizing animal models to assess potential human health risks [54,55,56,57,58]. Investigations into individual PFASs have revealed that the generation of reactive oxygen species (ROS) in HepG2 (a hepatoblastoma cell line) cells increases with the length of the PFAS carbon chain [58]. ROS generation is a critical marker of mitochondrial permeability transition, which subsequently leads to DNA damage and apoptosis [59,60,61,62]. However, research by Florentin et al. reported no significant ROS induction at PFOS concentrations of 300 μM or PFOA concentrations of 200 μM, suggesting that PFASs can elicit cytotoxicity through ROS-independent pathways [63]. Further in vitro studies are needed to elucidate the effects of structurally diverse PFASs on compound levels and cellular responses in HepG2 cells.

Additionally, Ojo et al. demonstrated that different PFAS combinations exhibit distinct cytotoxicity profiles in HepG2 cells. Notably, their findings revealed that mixtures containing PFOS predominantly displayed synergistic effects, whereas mixtures containing PFOA could exhibit either synergistic or antagonistic interactions depending on the composition [64]. The observed synergism may be attributed to increased cell membrane permeability, which amplifies cytotoxic effect [59,62]. Interestingly, PFASs with carboxylic acid functional groups showed greater agonistic potential compared to their sulfonic acid counterparts [56]. Despite these advancements, many fundamental questions remain regarding the interplay between individual PFASs, PFAS mixtures, and their hepatotoxic effects. Further research is imperative to unravel the mechanisms underlying these complex interactions.

Animal studies on PFAS exposure typically involve either sample collection from potentially contaminated environments or the design of controlled dosing regimens, exposure durations, and administration routes. Guruge et al. analyzed cow liver samples and reported that the PFOS ratios of fetal to maternal liver samples ranged from 0.26 to 1.2, suggesting that PFOS can cross the placental barrier during gestation and accumulate in the fetal liver [65]. Additionally, milk excretion in cows has been identified as a potential transmission pathway [66].

Mechanistic studies frequently employ rodent models. For instance, a significant increase in liver weight was observed in mouse pups exposed to 3.88 mg/kg PFOS [67]. Liver hypertrophy, along with signs of cell injury (elevated alanine aminotransferase and aspartate aminotransferase levels), was induced by PFOA administration at a dose of 5 mg/kg body weight. However, genotoxicity tests revealed no genotoxic effects in mouse liver tissue, indicating that PFASs are considered non-genotoxic [68]. The observed hepatotoxic effects are primarily attributed to the activation of the nuclear receptor peroxisome proliferator-activated receptor alpha (PPAR*α*) mediated by PFOA and PFOS at the molecular level [56,69].

Notable interspecies differences exist in the response to PFAS exposure. While PFOA and PFOS effectively activate PPAR*α* in rodent hepatocytes, they exhibit limited activation of this receptor in human hepatocytes [70]. Nevertheless, PFOA and PFOS disrupt lipid metabolism in human hepatocytes through their interaction with PPAR*α* [70]. Although extensive research has been conducted on PPAR*α* activation by PFOA and PFOS, the effects of other PFAS compounds on PPAR*α* activation warrant further investigation. Furthermore, other mechanisms may contribute to PFAS-induced hepatotoxicity and steatosis. These include activation of the constitutive androstane receptor (CAR), downregulation of nuclear factor erythroid 2-related factor 2 (NRF2), and upregulation of nuclear factor-kappa B (NF-κB) [71]. Recent studies have shown that emerging PFASs such as GenX and PFBS can also induce hepatocellular hypertrophy and lipid accumulation in rodent models, likely mediated through oxidative stress and dysregulation of fatty acid metabolism [72]. GenX exposure has been shown to induce steatosis and hepatomegaly in rodent models, with effects on genes involved in lipid metabolism and oxidative stress pathways [73].

### 2.2. Kidney Toxicity

Understanding the complex relationship between PFAS exposure and kidney function is essential for developing effective strategies to mitigate this public health challenge. Both animal and human studies have demonstrated that PFAS exposure significantly increases the risk of chronic kidney disease (CKD). This is likely because the kidneys, as the primary organ for filtering blood via the glomerulus, play a critical role in eliminating toxins from the body. PFAS accumulation in the kidneys can impair renal function, as evidenced by correlations between PFAS exposure and reduced glomerular filtration rates (GFR) [46]. GFR, clinically estimated (eGFR), is the most significant marker of kidney function for diagnosing kidney disease [74]. Healthy kidneys correspond to higher eGFR values, while a persistent eGFR below 60 mL/min/1.73 m^2^ is indicative of CKD [75]. Additionally, PFAS exposure has been linked to other chronic conditions such as obesity, diabetes, hyperlipidemia, hyperuricemia, and microvascular diseases that independently increase the risk of CKD [76].

A cohort study using National Health and Nutrition Examination Survey (NHANES) data from 2009 to 2010 identified a significant association between urinary PFAS concentrations and kidney function. The findings revealed that higher PFAS concentrations correlate with lower eGFR [74]. Occupational studies, such as those involving workers at the DuPont chemical plant, showed a slightly elevated CKD risk with PFOA exposure (3.11 standardized mortality ratio) [77]. Conversely, a study in China identified a stronger association between PFOA and CKD, suggesting that CKD severity may depend on the exposure level and duration [78].

PFAS exposure has also been linked to renal cell carcinoma (RCC) [38], the most common form of kidney cancer, accounting for 80 to 85% of all primary renal tumors [78]. A study measured pre-diagnostic serum concentrations of PFOA and seven other PFASs in 324 RCC cases and 324 controls. Results revealed a positive correlation between serum PFOA levels and RCC risk, implying that reduced kidney functionality may lead to PFOA accumulation and potentially elevate RCC risk [38]. While most studies focus on PFOA and PFOS, the Human Heredity and Health in Africa (H3Africa) epidemiology study highlighted the harmful effects of less common PFASs, such as perfluorononanoic acid (PFNA) and perfluorodecanoic acid (PFDA), on eGFR even at lower concentrations [79]. Interestingly, PFAS concentrations, including PFNA, PFOA, and PFOS, decrease by approximately 20% during pregnancy. This phenomenon is partially explained by plasma volume expansion, placental transfer, and increased GFR during pregnancy [80].

Recent studies have also explored PFAS mixtures’ impact on kidney function and lipid metabolism [80]. Chen et al. reported that PFASs, particularly PFOA, are associated with elevated uric acid levels but not with changes in GFR [81]. They also indicated that several lipid molecules mediate the relationship between PFASs and kidney dysfunction. In murine studies, PFOA and PFOS have been associated with glomerular hypertrophy and altered creatinine clearance, while GenX showed similar renal oxidative stress markers and mitochondrial disruption [82]. National studies have shown positive associations between PFAS exposure and hyperuricemia risk, with no identified safe threshold for PFAS exposure [83]. Patients with CKD using oral toxin adsorbents exhibited improved kidney function and reduced acrolein levels, suggesting potential links between decreased PFAS levels and improved renal outcomes [84]. Physiologically based in vitro-to-in vivo extrapolation (IVIVE) models accurately predicted renal clearance rates for PFASs, distinguishing substances with varying bioaccumulation risks based on their renal elimination characteristics [85]. This approach could prioritize PFASs with higher bioaccumulation potential for regulatory attention.

The association between PFAS exposure and chronic diseases, such as obesity, diabetes, hyperlipidemia, hyperuricemia, and microvascular conditions, has prompted research into its connection with CKD. One population study conducted over a 10-year period in the Dongfeng–Tongji cohort investigated the link between CKD with type 2 diabetes [86]. Li et al. analyzed baseline serum PFAS levels (PFOA and PFOS) among 967 diabetic patients. Their findings identified 267 CKD cases and revealed a negative association between PFOS and CKD incidence, particularly in patients with baseline eGFR levels below 70 mL/min/1.73 m^2^. Conversely, a separate study examined PFAS exposure and kidney function in 875 prediabetic adults over a 14-year period [76]. This research demonstrated that plasma PFAS concentrations during the Diabetes Prevention Program (1996–2002) were inversely associated with eGFR during follow-up (2002–2014), establishing that higher baseline PFAS levels in prediabetic individuals were significantly linked to lower eGFR over time.

PFAS exposure is also linked to elevated uric acid levels, known as hyperuricemia, which is associated with CKD [87]. The C8 Health Project in China specifically explored correlations between serum PFAS levels, primarily PFOA and PFOS, and uric acid levels in the general adult population. A notable finding from this study was the higher distribution of PFASs in participants with hyperuricemia, especially among men compared to women. Among the 20 PFASs measured, the branched PFOA isomer exhibited the strongest and most consistent associations with uric acid levels [88].

Moreover, these studies highlight broader implications of PFAS exposure on kidney function [74], providing evidence that PFASs not only disrupts renal performance but also contributes to other health complications. It is necessary for continued research and public health initiatives to further elucidate the impact of PFASs on kidney health and mitigate associated risks.

### 2.3. Cardiovascular Impacts

Cardiovascular toxicity encompasses an elevated risk of diseases such as heart attacks, strokes, hypertension, and disruptions in heart rhythm and function. Environmental contaminants like PFASs have been shown to interfere with various physiological processes critical to cardiovascular health. Evidence indicates that PFAS exposure contributes to platelet dysfunction, dyslipidemia, altered blood pressure regulation, and other mechanisms that promote the development of cardiovascular diseases.

A significant area of research involves the effects of PFASs on platelet cells, particularly their role in blood clot formation. Studies suggest that PFASs can accumulate in platelet cell membranes, disrupting calcium signaling and leading to heightened platelet activation. For example, exposure to PFOA was associated with increased platelet activation in response to thrombin receptor activator peptide 6 (TRAP-6), an agonist that stimulates platelet aggregation [89]. This heightened activation may impede blood flow to organs, increasing the risk of arterial thromboembolism [41]. Furthermore, research has demonstrated that elevated PFOS concentrations correlate with heightened platelet reactivity and altered platelet morphology, such as increased mean platelet volume and platelet distribution width. These changes amplify platelet aggregation, thereby raising the risk of cardiovascular disease [90].

Beyond platelet dysfunction, high cholesterol levels, a critical factor in limited blood flow and cardiovascular risk, have also been linked to PFAS mixture exposure. However, studies examining the relationship between PFASs and cholesterol levels have yielded mixed results. Research in children revealed a negative association between PFAS (mixture) concentrations and low-density lipoprotein cholesterol (LDL-C) but a positive association with high-density lipoprotein cholesterol (HDL-C) [91]. Conversely, another study identified a positive correlation between PFAS exposure and total cholesterol levels, including LDL-C [92]. Given the well-documented connections between LDL-C and coronary heart disease, cardiovascular mortality, and atherosclerotic disease, these findings underscore the intricate relationship between PFAS exposure and lipid metabolism in cardiovascular health [93].

PFAS exposure has also been investigated in relation to blood pressure regulation, but findings remain inconsistent. While some studies report positive associations between PFAS exposure and both systolic and diastolic blood pressure, others present conflicting evidence. For instance, higher PFOA exposure has been linked to elevated diabolic blood pressure in certain populations [94]. However, studies focusing on children and adolescents found no significant relationship between PFAS (PFOA, PFOS, and PFHxS) exposure and blood pressure [95]. Additionally, Pitter et al. reported that only 12.5% of individuals exposed to PFASs (PFOA, PFOS, PFHxS, and PFNA) exhibited hypertension [96].

Among pregnant women, elevated PFAS concentrations have been associated with increased systolic and diastolic blood pressure, though only a subset of these findings reached statistical significance. The connection between PFAS exposure and preeclampsia, a pregnancy complication characterized by high blood pressure, remains inconclusive. However, elevated blood pressure in pregnant women poses potential long-term cardiovascular risks for both the fetus and the mother [97]. Given the variability in results across studies, it is challenging to draw definitive conclusions about the impact of PFASs on blood pressure regulation. While several epidemiological studies have shown positive correlations between serum PFAS levels and elevated cholesterol (especially LDL-C), other studies reported null or even inverse associations. These discrepancies may arise from confounding factors, reverse causality, or differences in study populations and exposure windows. Overall, the preponderance of evidence suggests PFASs may impair lipid metabolism, but causality remains to be firmly established [98,99].

Further research has also explored the broader health implications of PFAS exposure, including its impact on fetal development. Ou et al. investigated the association between various PFASs and fetal growth. While acknowledging the need for larger sample sizes, they identified links between septal defects and 6m-PFOS (a branched perfluorooctane sulfonates), as well as between conotruncal defects and linear PFOS [100]. Additionally, PFASs’ effects on platelet cells and the circulatory system suggest potential cardiotoxicity. A 1997 study further examined the influence of PFASs on physical fitness and activity levels among third-grade students in Danish public schools. The findings revealed an inverse relationship between PFOA, PFDA, PFHxS, and leptin, a hormone critical for maintaining healthy body weight. Notably, PFHxS was inversely related to adiponectin in boys, while PFOA showed a positive correlation. These findings indicate that PFASs disrupt cell signaling pathways regulated by adipokines, potentially altering human metabolism [101]. Intriguingly, among individuals with diabetes, PFAS exposure exhibited an inverse association with coronary heart disease, as reflected in the odds ratios reported [102].

Interestingly, the risk of stroke did not exhibit a significant association with PFAS exposure, including specific compounds such as PFOS and PFHxS [89]. A study conducted between 2019 and 2020 documented the health outcomes of individuals potentially exposed to PFASs in areas under PFAS management. Across three regions, fewer than five cases of stroke were reported among hundreds of participants, suggesting minimal exposure-related risk [103]. Similarly, a population-based study of two Swedish cohorts concluded that PFAS exposure does not increase stroke risk and may even exhibit an inverse relationship [104]. Furthermore, PFASs could potentially influence blood pressure regulation due to its effect on oxygen transport [105]. Perfluorocarbons (PFCs), which share structural similarities with PFASs, demonstrate 25 times greater oxygen solubility than blood or water and maintain stability across temperature changes. Unlike hemoglobin, which shows reduced oxygen-carrying capacity over time, PFCs sustain a linear relationship with oxygen, potentially contributing to reduced hypertension [106].

A study investigating the cardiotoxicity of 56 PFAS compounds utilized human stem cell-derived cardiomyocytes from multiple donors, revealing significant inter-individual variability in responses [107]. Among these compounds, 46 were found to affect cardiac function, with several that pose notable cardiotoxic risks. In postmenopausal women, PFOA and PFOS exposure were associated with an elevated risk of cardiovascular diseases, likely mediated by inflammation-related proteins [108]. Animal models have further elucidated PFAS cardiotoxicity. In female rats, PFOS exposure impaired cardiovascular function, leading to increased blood pressure and decreased cardiac output. These effects were more pronounced in rats with intact ovaries [109], suggesting that PFOS influences cardiac function via hormone-dependent mechanisms, while vascular dysfunction occurs independently of ovarian status. Zebrafish studies also highlighted the developmental cardiotoxicity of perfluoroalkyl acids (PFAAs), with PFOA exhibiting the highest toxicity [110]. PFAAs disrupted cardiac muscle contraction and heart development more severely than their salt counterparts.

The body of evidence highlights the complex relationship between PFAS exposure and cardiovascular health, encompassing mechanisms such as platelet dysfunction, dyslipidemia, and blood pressure regulation. Although PFAS exposure has been linked to various cardiovascular risk factors, its definitive impact on cardiovascular diseases, including stroke, remains inconclusive. Further research is needed to understand the mechanisms driving PFAS-induced cardiovascular effects and to determine its long-term impact on cardiovascular outcomes. Table 1 shows a summary of common experimental models utilized to assess PFAS toxicity.

## 3. Neurotoxicity

It is known already that PFASs can enter the human body via inhalation, ingestion, and dermal contact. A key concern lies in their ability to traverse biological barriers, such as the blood–brain barrier (BBB) which raises significant questions about their impact on neurological function. Understanding the neurotoxic potential of PFASs is also essential for developing strategies to mitigate their effects on human health, especially mental health.

Studies have reported median PFAS concentrations in human serum ranging from 0.01 to 10,400 ng/mL [31]. While primarily detected in the liver and blood, PFASs have also been identified in the brain, with higher accumulation observed in regions such as the hippocampus, hypothalamus, medulla, thalamus, and brainstem [116]. The precise mechanisms by which PFASs penetrate the brain remain unclear, but proposed pathways include binding to transporters and disassembly of the BBB. For example, PFOS induces the production of ROS, which remodel actin filaments and increase endothelial permeability. Additionally, compounds like PFOA, PFNA, PFDA, and PFHxS inhibit P-glycoprotein (P-gp), reducing the brain’s ability to expel xenobiotics such as PFASs [117]. Notably, long-chain perfluoroalkyl carboxylic acids (PFCAs, C10-C15) may cross the BBB through mechanisms resembling fatty acid transport, explaining their higher concentrations in the brain [116]. Investigating these pathways is critical for devising strategies to protect neurological health.

In rats, PFOA exposure increased the number of processes and branches per cell at concentrations of 1 and 10 mM, while reducing cell body area at 1–100 mM [118]. PFASs have also been implicated in studies on dopamine regulation. In humans exposed to PFAS-contaminated water in northern Italy, significant accumulation of PFOA, PFHxS, and perfluorohexanoic acid (PFHxA) in the brain was linked to dopaminergic degeneration [119]. Similarly, in mice, PFAS exposure reduced tyrosine hydroxylase expression and D2 dopamine receptor levels, potentially explaining the observed increase in attention-deficit/hyperactivity disorder (ADHD) frequency among exposed populations [120]. In *Caenorhabditis elegans*, PFOS induced dopaminergic neurotoxicity at concentrations as low as 75 mg/L (150 µM) after 48 h, leading to behavioral abnormalities such as increased repulsion time [121]. However, the precise impact of PFASs on the brain remains unclear. For instance, studies on *Ursus maritimus* (polar bears), whose physiology resembles that of humans, suggest differential effects of PFOS on muscarinic acetylcholine receptors between the cerebellum and frontal cortex, indicating region-specific responses to PFASs. Emerging PFASs such as GenX and PFBS have been shown to cross the blood–brain barrier and induce neuroinflammatory markers, including elevated IL-6 and glial fibrillary acidic protein (GFAP), in zebrafish models [122]. PFBS, despite its shorter half-life, demonstrated significant neurotoxic effects by elevating reactive oxygen species and modulating cholinergic signaling in neuronal models [123].

PFASs have also been shown to inhibit neurite outgrowth in neuronal cells at environmentally relevant concentrations [124], suggesting that both individual PFASs and their mixtures may impair neuronal development. Long-term neurotoxic effects of early-life PFOA exposure include persistent neuronal damage, altered calcium activity, and transcriptomic changes linked to Alzheimer’s disease [125]. Obiako et al. further demonstrated that both long-chain (PFOA) and short-chain (perfluorobutanoic acid (PFBA)) PFASs induce oxidative stress and disrupt metabolic processes in neuronal cells, with PFBA exerting more pronounced effects [126]. The oxidative damage and metabolic dysfunction caused by PFASs are likely critical pathways in their neurotoxicity. Additionally, studies on the gut–brain axis revealed that PFHxS exposure alters gut microbiota composition, potentially mediating neurobehavioral issues such as conduct problems in children [127].

While PFAS exposure has been associated with neurotoxicity, dopaminergic degeneration, and neurodevelopmental impacts, further research is necessary to fully elucidate these effects and to develop comprehensive preventive measures to safeguard human health.

## 4. Immunotoxicity

Given that the gut microbiota–brain axis plays a pivotal role in modulating immune responses, it is plausible that PFAS-induced disruptions in gut microbial balance may contribute to downstream immunotoxic effects, including altered cytokine production and reduced mucosal immunity [128].

Research on immunotoxicity has shown that PFAS exposure disrupts various aspects of immune function in both vertebrate animals and humans. Alterations in inflammatory cytokines, which play crucial roles in regulating immune responses and inflammation, have been widely documented. Proteomic studies have linked PFAS exposure to significant changes in inflammatory markers, including cytokines and chemokines that mediate the activity of macrophages, T cells, and other immune cells. These changes affect processes such as neuronal regulation, angiogenesis, and anti-infection responses [129].

Numerous vertebrate animal studies have demonstrated the immunotoxic effects of PFASs [130,131,132,133]. McDonough et al. reported that PFOA and PFOS caused adverse immune outcomes in male and female C57BL/6 mice, including reduced body weights and antigen-specific antibody production (15% and 13% decreases, respectively). Conversely, relative liver weights increased by up to 200%, accompanied by a 12-fold rise in liver peroxisome proliferation. However, other studies found that newer PFAS compounds, such as perfluoromethyloctanoic acid (PFMOAA) and perfluoromethylopropionic acid (PFMOPrA), did not significantly alter natural killer (NK) cell activity or T-cell-dependent antibody response (TDAR) at high administration doses of up to 50 mg/kg. These compounds also showed no statistically significant changes in immune cell populations [130]. Additionally, dermal exposure to PFOA caused immunotoxic effects, including spleen and thymus weight decreases (0.5–2%) and liver weight increases, along with changes in splenic B-cell numbers [131]. Using female B6C3F1 mice, De Guise et al. demonstrated altered Th1/Th2 and pro-inflammatory cytokine levels following PFOA exposure and keyhole limpet hemocyanin (KLH) stimulation, suggesting reduced IgM response mediated by lower Th2 cytokine levels [132].

Zebrafish has also been employed to investigate PFAS immunotoxicity [128,134,135]. Studies have shown that PFASs and their alternatives, such as 6:2 chlorinated polyfluorinated ether sulfonate (F-53B) and sodium p-perfluorous nonenoxybenzene sulfonate (OBS), can induce anti-inflammatory effects in zebrafish liver, alter kidney and intestinal histopathologies, and disrupt intestinal microbiota composition. Zebrafish larvae were found to be more susceptible to F-53B than embryos, with estimated 96-h LC50 (Lethal concentration 50) values of 2.4 mg/L and 15.1 mg/L, respectively. Excessive production of nitric oxide (NO) and reactive oxygen species (ROS), coupled with dysregulated immune-related gene expression, further exacerbated immunotoxicity in larvae [134]. PFOS was shown to activate the TLR/myd88/P65 pathway, suppress IFN and BAFF mRNA expression, and disrupt lipid metabolism, intensifying its immunotoxic effects [135]. Similar immune system toxicity has been reported in other vertebrates, including bass (*Morone saxatilis*), American alligator (*Alligator mississippiensis*), and perch (*Perca fluviatilis*) [136,137,138].

Human studies have also demonstrated the detrimental effects of PFASs on immune health. Barton et al. noted that higher concentrations of multiple PFASs were significantly associated with lower levels of interleukin-1β (IL-1β). Perfluoroalkyl carboxylic acids (PFCAs) showed inverse associations with tumor necrosis factor alpha (TNF-α), while perfluoroalkyl sulfonic acids (PFSAs) exhibited positive correlations [139]. Bamai et al. linked prenatal exposure to long-chain PFASs (C8 and above) to an increased prevalence of infectious and allergic diseases, such as respiratory syncytial virus (RSV) and pneumonia, in children under 7 years old. In adults, elevated serum PFAS concentrations have been associated with altered inflammatory profiles due to exposure through contaminated drinking water [139]. A study in Mid-Ohio Valley linked PFOA contamination to increased lymphocyte counts, particularly total lymphocytes [140]. Among the elderly, higher plasma PFAS levels correlated with decreased levels of inflammatory markers such as hepatocyte growth factor (HGF) and colony-stimulating factor 1 (CSF-1) [141].

Advances in computational toxicology have enabled the simulation of PFAS effects on the immune system [142]. Pappalardo et al. utilized the Universal Immune System Simulator for Toxicants (UISS-TOX) to model PFAS interactions with the immune system, predicting outcomes such as reduced antibody production and diminished vaccine responses based on PFOS/PFOA plasma concentrations. This approach offers insights into the immunotoxic potential of PFASs and its broader implications.

The COVID-19 pandemic has further underscored the impact of PFASs on immune health. Immune suppression from PFAS exposure may exacerbate severe acute respiratory syndrome coronavirus 2 (SARS-CoV-2) symptoms [143]. Perfluorobutanoic acid (PFBA), known to accumulate in the lungs, has been associated with severe COVID-19 outcomes, irrespective of age, sex, or comorbidities [144].

Studies in both animal models and humans indicate that PFAS exposure disrupts immune cell populations, impairs antibody production, and alters cytokine expression. These effects include liver peroxisome proliferation, changes in immune phenotypes, and increased susceptibility to infectious diseases, particularly in children. Recent data suggest that newer PFASs like PFHxS may also contribute to suppressed vaccine response and immunoglobulin dysregulation in pediatric populations [145]. In human biomonitoring studies, PFHxS levels correlated with lower antibody titers post-vaccination, suggesting functional immunosuppression even at low concentrations [146].

### Co-Exposure Toxicology with Other Contaminants

Emerging studies suggest that co-exposure to PFASs and other environmental pollutants, such as heavy metals (e.g., cadmium), pesticides (e.g., chlorpyrifos), and mycotoxins, may result in synergistic or antagonistic toxic effects. For instance, mice exposed to PFOS and cadmium displayed amplified hepatic inflammation and altered cytokine profiles [147]. Moreover, in vitro co-exposure to GenX and aflatoxin B1 resulted in exacerbated mitochondrial stress and reduced cell viability [148]. These findings highlight the complexity of risk assessment in real-world environmental contexts. Table 2 provides a comprehensive summary of selected PFAS toxicity, highlighting affected organs, underlying mechanisms, and key pathways covered in this review.

## 5. Conclusions

Understanding the toxicity of PFASs on human health is crucial for developing effective strategies to mitigate pollution and safeguard public well-being. The extensive body of research on PFASs and their associated adverse health effects, as outlined in this review and the broader scientific literature, underscores the urgent need for precautionary measures. Despite this progress, further research is required to employ innovative methodologies and rigorously designed studies that deepen our understanding of PFAS-related health effects. These studies should aim to refine risk assessment strategies for this diverse chemical family while elucidating the mechanisms underlying PFAS toxicity. Additionally, identifying reliable biomarkers of exposure and susceptibility remains a critical goal to enable earlier detection and targeted interventions. Filling these knowledge gaps will bolster efforts to protect public health and guide regulatory actions aimed at minimizing the harmful impacts of PFASs. Regulatory measures and public health interventions are essential to reduce PFAS exposure and ensure the safety of populations worldwide. Such actions will be pivotal in mitigating the risks posed by these persistent contaminants and promoting long-term environmental and human health.

Future research should focus on the long-term effects of emerging PFASs, explore real-world co-exposures with other environmental contaminants (such as pesticides and heavy metals), and adopt integrative tools like high-throughput screening, toxicogenomics, and AI-driven toxicological modeling [149,150,151]. Expanding human cohort studies and developing improved in vitro platforms [152] such as organoids and organ-on-chip systems will further bridge the gap between animal models and human relevance, supporting more accurate risk assessments.

## Figures and Tables

**Table 1:** Common experimental models used to evaluate PFAS toxicity.

Model Type	System	Application	Example Compounds Studied	Key References
In vitro	HepG2 cells	Mechanistic insight into ROS production; apoptosis	PFOS, GenX, and PFBS	A
In vivo (rodents)	Mouse/Rat	Systemic toxicity; liver/kidney effects	PFOA, PFOS, and GenX	B
In vivo (zebrafish)	Zebrafish embryos	Developmental and immune effects and neurotoxicity	PFOS, F-53B, and OBS	C

Footnote: In vitro systems such as HepG2 offer mechanistic insights, whereas in vivo rodent and zebrafish models help understand systemic and developmental toxicity. Corresponding references: A [64,111], B [112,113], and C [114,115].

**Table 2:** Summary of PFAS toxicity, affected organs, mechanisms, and key pathways.

PFAS	Organ	Mechanisms/Modes of Action	Key Pathways/Effects	References
PFOS, PFOA	Liver	Cellular injury, ROS-independent cytotoxicity, DNA damage, and apoptosis	Activation of PPARα, CAR; downregulation of NRF2	A
GenX	Liver	Mediated through oxidative stress and dysregulation of fatty acid metabolism	Induce hepatocellular hypertrophy and lipid accumulation	B
PFOS, PFOA	Kidney	Decreased GFR, associated with CKD and RCC	Serum concentrations linked to renal cell carcinoma risk	C
PFNA, PFDA, PFOS	Kidney	Reduced eGFR significantly	Impaired renal function	D
PFOS, PFOA	Cardiovascular system	Platelet dysfunction, dyslipidemia, and altered blood pressure regulation	Increased platelet activation; elevated cholesterol; mixed results on blood pressure	E
PFHxS, PFHxA	Brain	Neurotoxic effects, increased ROS, and cholinergic signaling modulation	Dopaminergic degeneration	F
GenX, PFBS	Brain	Induce neuroinflammatory markers, including elevated IL-6 and glial fibrillary acidic protein (GFAP)	Disruption of dopamine regulation	G
PFOS	Immune system	Altered Th1/Th2 and pro-inflammatory cytokine levels and keyhole limpet hemocyanin (KLH) stimulation	Spleen and thymus weight decreases and liver weight increases, along with changes in splenic B-cell numbers	H

Footnote: the corresponding references appear in the table: A [59,60,61,62,63,68,69,70,71], B [72,73], C [38,46,77,79,81,88], D [79,80], E [89,90,91,92,94,95,96], F [119,120], G [122], and H [130,131,132].

## Data Availability

Data sharing is not applicable.
